# Reliability of MUSE 2 and Tobii Pro Nano at capturing mobile application users' real-time cognitive workload changes

**DOI:** 10.3389/fnins.2022.1011475

**Published:** 2022-11-28

**Authors:** Limin Zhang, Hong Cui

**Affiliations:** ^1^China School of Fine Arts, Huaiyin Normal University, Huaian, China; ^2^USA School of Information, University of Arizona, Tucson, AZ, United States

**Keywords:** cognitive workload, EEG, eye tracking, eye movement, GUI

## Abstract

**Introduction:**

Despite the importance of cognitive workload in examining the usability of smartphone applications and the popularity of smartphone usage globally, cognitive workload as one attribute of usability tends to be overlooked in Human-Computer Interaction (HCI) studies. Moreover, limited studies that have examined the cognitive workload aspect often measured some summative workloads using subjective measures (e.g., questionnaires). A significant limitation of subjective measures is that they can only assess the overall, subject-perceived cognitive workload after the procedures/tasks have been completed. Such measurements do not reflect the real-time workload fluctuation during the procedures. The reliability of some devices on a smartphone setting has not been thoroughly evaluated.

**Methods:**

This study used mixed methods to empirically study the reliability of an eye-tracking device (i.e., Tobii Pro Nano) and a low-cost electroencephalogram (EEG) device (i.e., MUSE 2) for detecting real-time cognitive workload changes during N-back tasks.

**Results:**

Results suggest that the EEG measurements collected by MUSE 2 are not very useful as indicators of cognitive workload changes in our setting, eye movement measurements collected by Tobii Pro Nano with mobile testing accessory are useful for monitoring cognitive workload fluctuations and tracking down interface design issues in a smartphone setting, and more specifically, the maximum pupil diameter is the preeminent indicator of cognitive workload surges.

**Discussion:**

In conclusion, the pupil diameter measure combined with other subjective ratings would provide a comprehensive user experience assessment of mobile applications. They can also be used to verify the successfulness of a user interface design solution in improving user experience.

## Introduction

Portable media devices, such as smartphones, have become an increasingly pervasive part of our lives. In 2020, the number of smartphone users in the United States was estimated to reach 294.15 million and will reach 311.53 million by 2025 (O'Dea, 2021). American adults spent around 3 h and 30 min per day using mobile phones in 2019, with an increase of about 20 min from 2018, according to Zenith (Molla, 2020). Correspondingly, the number of applications in the App Store has soared from the initial 500 in 2008 to roughly 2.22 million available applications in 2021 (Ceci, 2022). As a result, mobile phone applications receive greater attention from the Human–Computer Interaction (HCI) field, resulting in a surge in the number of publications. We input a query “usability AND phone AND application” with custom time ranges: 1991–2000, 2001–2010, and 2011–2020 in Google Scholar, and get 8,500, 55,500, and 68,200 results.

Researchers in Human–Computer Interaction (HCI) fields have long recognized usability as the core of product design, including the application design of smartphones (Shneiderman, 1986; Nielsen, 1993; Brooke, 1996; Dumas et al., 1999). Previous research has manifested that cognitive workload is an essential aspect of product usability (Harrison et al., 2013; Davids et al., 2015).

Measuring cognitive workload has been recognized as one challenge when taking objectivity and causality into consideration (Brunken et al., 2003; Brünken et al., 2010). Instruments such as the NASA questionnaire (Hart and Staveland, 1988) help solicit *perceived* cognitive workload from users *after* a task is completed. Results obtained through such instruments are tinted with a level of subjectivity and put the causality between stimuli and reported cognitive workload in question.

On the other hand, electroencephalogram (EEG) devices can objectively monitor and record the brain's electrical activities and researchers have successfully identified signals from EEG to measure cognitive workload (Gevins and Smith, 2003; Antonenko et al., 2010a; Makransky et al., 2019). And eye movement data have been collected and analyzed to guide and advise various aspects of product design: navigation, page layout, user interface (UI) visualization style with design elements, advertisement, user viewing behaviors, and user cognitive workload (Goldberg and Wichansky, 2003; Nielsen and Pernice, 2010).

However, most of the studies were not executed in a smartphone setting and they cannot provide direct evidence for the reliability of EEG and eye-tracking devices to measure cognitive workload in a smartphone setting, due to several variabilities between desktop/laptop computer settings and smartphone settings. The screen sizes of desktop/laptop computers and smartphones are different: large vs. small. Users' interactions with these devices are distinct: cursors vs. gestures. The content compositions are not the same either: columns vs. scrolling. Physically, the users interact with their smartphones in different manners, such as: (1) one-handed, (2) two-handed, and (3) cradled, (4) no-handed; and in three body postures: walking, standing, and sitting/lying (Hoober, 2013).

Based on a thorough review of the related literature, we have identified three gaps as follows:

1. Despite the significance of cognitive workload, it tends to be overlooked in the HCI field (Zhang and Adipat, 2005; Coursaris and Kim, 2006; Harrison et al., 2013).
2. The majority of studies we reviewed only examine the overall cognitive workload during tasks and fail to study the instantaneous or peak cognitive workload during tasks and its relationship with product interface design and usability.
3. There is little direct evidence to suggest that EEG and eye-tracking devices are reliable in measuring cognitive workload in a smartphone setting.

To address these gaps, we need to answer the two questions first:

1. Are EEG data collected by MUSE 2 and eye movement data recorded by Tobii Pro Nano valid, reliable, and feasible as assessment tools for the real-time cognitive workload?
2. Are measures collected by the two devices (averages of Event-related (de-)synchronization (ERD) of Alpha, Beta, and Event-related synchronization (ERS) of Theta for TP9, TP10, AF7, and AF8; pupil dilation, saccade duration and saccade number, fixation duration, and fixation number) sensitive to the cognitive workload of different N-back tasks in real time when the tasks are completed on a smartphone?

To answer the questions asked above, we employed a low-cost and portable electroencephalogram (EEG) device (MUSE 2, https://choosemuse.com/muse-2-guided-bundle/) and a user-friendly eye-tracking device (Tobii Pro Nano, https://www.tobiipro.com/product-listing/nano/) to detect real-time cognitive workload changes during N-back tasks on a smartphone. Our hypotheses were simple—we predict that the EEG device, MUSE 2, and the eye tracker device, Tobii Pro Nano with smartphone adopters, are reliably quantifying the cognitive workload of users performing tasks on a smartphone by these measures listed above.

## Background

### Cognitive workload in usability

According to the latest ISO 9241-11 (2018), usability is “the extent to which a system, product, service can be used by specified users to achieve specified goals with effectiveness, efficiency, and satisfaction in a specified context of use.” Various standards and models list a range of attributes for usability. Among these attributes, the cognitive workload is defined by Bevan and MacLeod (1994) as the mental effort required to perform tasks and is particularly important in safety-critical applications. It refers to the user's cognitive processing amount to using the application (Harrison et al., 2013).

### Cognitive workload measurements in HCI

The cognitive workload measurements can be roughly grouped into three broad categories: subjective self-assessment rating scales, performance measures, and psychophysiological measures (Wilson and Eggemeier, 1991; Cain, 2007; Evans and Fendley, 2017). Here, we only introduce two measures adopted in this research: electroencephalogram (EEG) and eye movement in psychophysiological measures.

#### Measurement of cognitive workload using electroencephalogram

Electroencephalogram (EEG) is an electrophysiological method of monitoring and recording the brain's electrical activity. Most of the time, an EEG device that comprises non-invasive electrodes is placed along a subject's scalp. These electrodes capture voltage fluctuations resulting from ionic currents within the brain's neurons.

In recent years, researchers have been evaluating the potential of the EEG as a measure of cognitive workload in different task conditions: arithmetic tasks (Anderson et al., 2011; Cirett Galán and Beal, 2012; Kumar and Kumar, 2016; Borys et al., 2017; Chin et al., 2018); cognitive tasks (Trammell et al., 2017); reading tasks (Dimigen et al., 2011; Knoll et al., 2011; Gwizdka et al., 2017); listening to music tasks (Asif et al., 2019); visual search task (Winslow et al., 2013; Hild et al., 2014); learning tasks (Dan and Reiner, 2017; Mazher et al., 2017; Notaro and Diamond, 2018); and vehicle driving task (Cernea et al., 2012). These studies confirm the fact that EEG provides reliable signals for studying cognitive workload in their respective settings.

Event-related (de-)synchronization (ERD/ERS) with Alpha, Theta, and Beta bands is one of the three most popular analysis techniques (Cabañero et al., 2019). Event-related (de)synchronization (ERD) is a recognized rate-of-change metric for oscillatory EEG dynamics, which was originally developed to quantify changes in the Alpha band (Pfurtscheller and Aranibar, 1977). Synchronization is a process where neurons are getting in line (synchronized) to enter an idling state. Desynchronization is a process where individual neurons get ready to perform their parts in a task. The steps of performing a task are: neurons desynchronize (wake up), perform tasks, and neurons synchronize (rest).

To obtain percentage values for ERD/ERS, the power within the frequency band of interest in the period after the event is given by A, whereas that of the preceding baseline or reference period is given by R. The percentage decrease (or increase) from the reference interval (R) to the activation interval (A) (before responding) was defined as


(1) $$\begin{array}{l} ERD/ERS\%=\left[\left(R--A\right)/R\right]*100\% \end{array}$$

(Pfurtscheller and Aranibar, 1977; Pfurtscheller and Lopes da Silva, 1999; Pfurtscheller, 2001).

Negative values computed by Equation 1 indicate power increase and desynchronization (ERD), and positive values indicate power decrease and synchronization (ERS).

Pfurtscheller and Lopes da Silva (1999) recommended that the term ERD is meaningful only if the baseline measured some seconds before the event represents rhythmicity seen as a clear peak in the power spectrum. Similarly, the term ERS only has a meaning if the event results in the appearance of a rhythmic component and therefore in a spectral peak that was initially not detectable (Pfurtscheller and Lopes da Silva, 1999).

The quantification of ERD/ERS was divided into four steps, first, the bandpass filtering was carried out for all Event-related trials; second, the amplitude samples were squared to obtain the power samples; third, the power samples of all trials were averaged; and fourth, the time samples were averaged to make the data smooth and reduce (Pfurtscheller and Lopes da Silva, 1999).

The review articles (Klimesch, 1999; Antonenko and Niederhauser, 2010b) concluded that with increasing task demands Theta synchronizes (decreases), whereas Alpha and Beta desynchronize (increase) (Pfurtscheller and Berghold, 1989; Neubauer and Fink, 2003; Stipacek et al., 2003; Klimesch et al., 2005; Neubauer et al., 2006; Scharinger et al., 2016; Saitis et al., 2018).

#### Measuring cognitive workload using eye movement data

Multiple kinds of eye movement data related to cognitive workload can be reliably collected using a high-quality eye-tracking device.

Pupil dilation is an involuntary response, in which the pupil diameter changes to protect the retina or to respond to a shift in fixation between objects at different distances. Previous research has shown that users' pupils dilate when the difficulty of the task increases and more cognitive effort has been allocated to solve the task (Granholm et al., 1996; Pomplun and Sunkara, 2003; Klingner et al., 2008; Chen et al., 2011; Porta et al., 2012; Rafiqi et al., 2015; Gavas et al., 2017; Ehlers, 2020). Accounting for individual and environmental differences, it is necessary to measure pupil diameters while referencing an adaptive baseline (Lallé et al., 2016).

According to Purves et al. (2001), saccades are rapid and ballistic movements of eyes that change the fixations abruptly. Previous research has found that growth in saccade velocity indicates a greater task difficulty (Barrios et al., 2004; Chen et al., 2011; Lallé et al., 2016; Zagermann et al., 2018).

Eye fixation refers to a focused state when eyes dwell voluntarily over some time and is the most common type of eye-tracking event (Zagermann et al., 2016). Previous research has proven that the correlation between the duration of fixation and the cognitive processing level is positive (Rudmann et al., 2003; Goldberg and Helfman, 2010; Chen et al., 2011; Wang et al., 2014; Zagermann et al., 2018).

### Devices in measuring cognitive workload

As previously reviewed literature shows, the measures of computing from EEG and eye movement data have been proven to be effective for detecting cognitive workload changes. However, most of the studies were conducted in smartphone settings, and the devices adopted in these studies are not suitable for use in smartphone usability testing environment.

Grateful to technology development, there are a wide range of choices in the selection of devices to capture the EEG data and eye-tracking data, respectively. Some examples of the EEG devices, ordered at prices, from low to high include: MUSE 2 headband, Emotiv Insight, OpenBCI, ANT Neuro, BioSemi, etc. (Farnsworth, 2019). A ranking of the top eye-tracking companies, ordered by the number of publications found through Google Scholar, is Tobii, SMI, EyeLink, Smart Eye, LC Technologies, Gazepoint, The Eye Tribe, etc. (Farnsworth, 2020).

**Figure 1:**
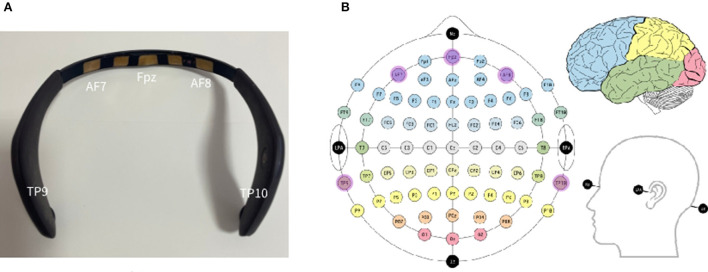
**(A)** MUSE 2 EEG headband; **(B)** EEG electrode positions in the 10-10 system using modified combinatorial nomenclature, along with the fiducials and associated lobes of the brain. Adopted from https://en.wikipedia.org/wiki/10%E2%80%9320_system_%28EEG%29#/media/File:EEG_10-10_system_with_additional_information.svg..

Among listed choices, the MUSE 2 headband (Figure 1A, $250) is an easy-to-use, affordable, and portable EEG recording system from InteraXon Inc. It is a four-channel headband with dry electrodes at positions AF7, AF8, TP9, and TP10 (Figure 1B). The headband is connected to the app on phone via Bluetooth, which makes it a great tool for detecting cognitive workload while the user is performing the task on smartphones, especially in some field experiments, of course, after its reliability is verified.

Despite the small number of sensors and the mismatch in the locations of the sensors to the standard 10–20 electrode positioning system, several studies have shown that the MUSE headband has the potential to provide good quality EEG data. Two studies (Arsalan et al., 2019; Asif et al., 2019) adopted MUSE 2 to capture EEG data and adopted classifiers to classify stress levels. Another study (Papakostas et al., 2017) also adopted MUSE EEG to predict the user task performance, and they achieved a maximum accuracy rate of 74%. Krigolson et al. (2017) collected data by MUSE EEG system, and the results showed quantifiable N200 and P300 Event-related potential (ERP) components in the visual oddball task and the reward positivity. However, these studies cannot provide direct evidence on the EEG data captured by the MUSE EEG system is reliable for cognitive workload changes.

Some studies have pointed out MUSE's limitations. Ratti et al. (2017) compared two medical grade (B-Alert, Enobio) and two consumer (MUSE, Mindware) EEG systems in five healthy subjects. Results showed that EEG data can be successfully collected from four devices, yet MUSE showed a broadband increase in power spectra and the highest relative variation across test–retest acquisitions. Another study has also shown that the data collected by MUSE headband were of poor quality under noisy conditions, such as at a public lecture (Przegalinska et al., 2018). To explore MUSE 2's potential as a great tool in smartphone usability testing, we still need direct empirical evidence on the reliability of MUSE 2 in capturing EEG data for measuring cognitive workload.

**Figure 2:**
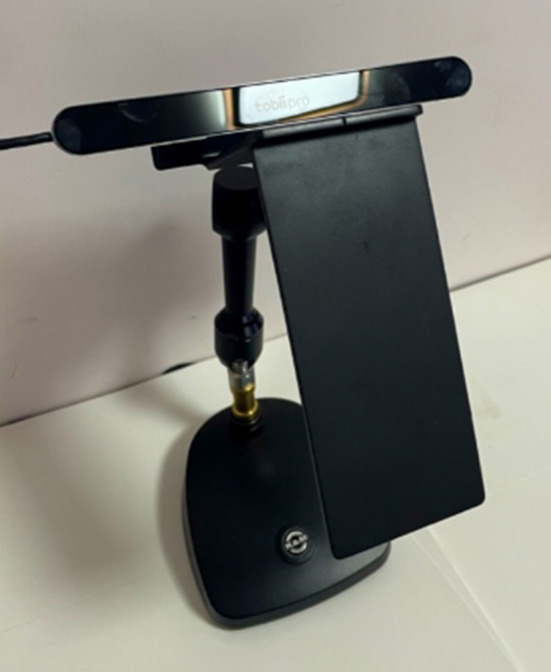
Tobii Pro Nano with mobile testing accessory.

Having picked an EEG device with its usefulness still under investigation, we selected a well-established eye-tracking device for this study to control the risk. We chose Tobii Pro Nano because it is one of the top eye-tracking companies and has been used in 20.5 k publications. It is also an accessible and efficient approach to capturing eye movement (Figure 2) and is used by many HCI researchers (Sugaya, 2019; Ehlers, 2020; Lee and Chenkin, 2020). Ehlers (2020) adopted Tobii Pro Nano to capture the pupil diameter and confirmed that it is a valid indicator of cognitive workload. Lee and Chenkin (2020) evaluated Tobii Pro Nano's potential to differentiate between experts and novices in the interpretation of POCUS clips in medical fields. Sugaya (2019) used Tobii Pro Nano to test an assumption about the meaning-making process of adjective expression formation.

Tobii Pro Nano can be mounted on a mobile testing accessory, also manufactured by Tobii (Figure 2). It has a screen capture device (Figure 2) connecting directly to smartphones. The screen capture device records a high-definition (HD) video of the mobile device's screen at 60 frames per second with a latency of only 10 milliseconds (Mobile Testing Accessory | Perfect for Usability Tests., 2020). Yet, the mobile testing accessory is just in the market, with no research done on it. The other great device for smartphone experiments is Tobii Pro Glass with a much higher price. If we can provide a piece of evidence on the reliability of Tobii Pro Nano with a mobile testing accessory, it could be a high-performance cost ratio choice for researchers.

## Methods

The goal of the experiment was to examine the reliability of the MUSE 2 headband and Tobii Pro Nano with a mobile testing accessory for detecting cognitive workload changes during a smartphone task and to select the best measure(s) computed from data collected by the two devices. The measures adopted in the experiment are (1) ERD percentage for Alpha, Beta, and ERS Theta rhythms extracted from EEG data; (2) multiple eye movements: pupil dilation, saccade duration, saccade number in second, fixation duration, and fixation number in second; and (3) user performance data: reaction time and accuracy rate.

### Participants

This study was approved by the UA IRB office (Protocol Number: 2101428836) and obtained permission from Qinghe High School, Jiangsu, China.

We recruited 5 students as pilots and 30 students as participants from Qinghe High School, Jiangsu, China. The inclusion criteria were normal vision or correct to normal vision, normal cognitive function, and proficiency in smartphones.

Students who participated either as pilots or as participants were compensated 50 in Chinese currency after they complete the task successfully.

### Apparatus

We used the MUSE 2 headband and Tobii Pro Nano as the devices to collect cognitive workload-related measures.

The environment's brightness variations produce changes in the pupil size (Pfleging et al., 2016; Zagermann et al., 2016). Therefore, the experiment was conducted in a room with lightproof curtains down to avoid natural lighting conditions, and electric lights on the room ceiling created a consistent lamination for the experiment. Environment, such as noise, also impacts cognitive load (Örün and Akbulut, 2019). We made sure the experiment room was free of all noise during the experimental sessions. All devices were sanitized before the next participant came.

When using EEG devices, one has to fulfill several other requirements. These include a clean scalp, clean electrodes, minimum participant activities, including head movements, since a small movement could generate muscle-based signals known as artifacts (Pratama et al., 2020). We instructed all participants to stay as still as possible and not to wear any makeup during the experiment. Also, as the electrodes need to be attached to the back of the ears, we encouraged the participants to wear contacts instead of glasses. We also provided a disposable wet cloth for participants to moisturize their foreheads and back of the ears to get a better connection of the EEG headset.

### Task: N-back task

*N*-back tasks are continuous-recognition measures that present stimulus sequences, such as letters or pictures. A sequence of stimuli is presented to the participants one by one. The participants are required to make a decision as to whether the current stimulus is the same as the one presented in N trials ago (Coulacoglou and Saklofske, 2017). The N can be 0, 1, 2, 3, etc. There is an increase in difficulty in tasks while N increases. An N-back task is a useful tool for experimental research on working memory (Jaeggi et al., 2010), and it has been adopted to manipulate cognitive workload (Reimer et al., 2009; Ayaz et al., 2010; Yokota and Naruse, 2015).

**Figure 3:**
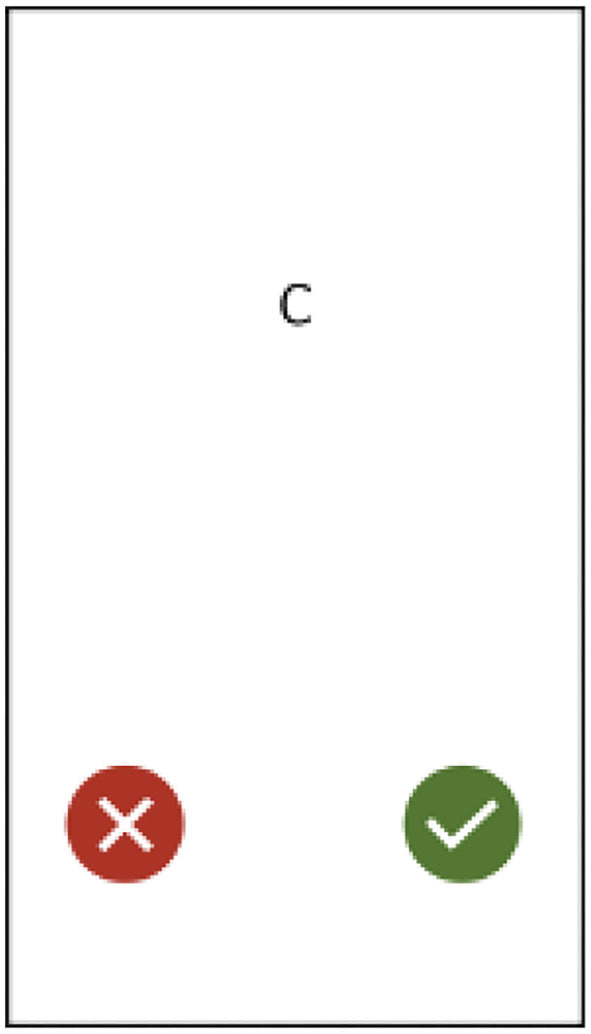
A screenshot of the N-back task.

In this study, all participants completed an N-back task. When employed in a computer setting, the participants of the experiment can press individual keys on keyboards as “YES” or “NO”. To cope with the touch screen of a smartphone, we placed “ × ” on the left bottom corner, and “√” on the right bottom corner of the smartphone screen (Figure 3).

In this study, we employed a 1 back task and a 2 back task to create a low cognitive workload condition and a high cognitive workload condition. The rationale of only including 1 and 2 levels is to simulate cognitive workload levels that smartphone users would experience in the real world.

The key features of the N-back task implementation were:

- Four sets of letters were created and arranged in two groups for a training block and an experiment block.
  ° Training Block:
    * Five trails of one back task (EEIPP) as a training session,
    * Six trails of two back task (OSOMLI) as a training session;
  ° Experiment Block:
    * low cognitive workload block: 20 trials of 1 back task (DAABEEDRRODHHRDSSELDD);
    * high cognitive workload block: 21 trails of 2 back (BAEAAEASHSAELEOBBBOSHS).
- These two sets of letters in the experiment block were designed to have an identical “YES” or “NO” response sequential: YNNNYNNNYNNNYNNYNNYN.
- Each stimulus was presented for maximally 3,000 milliseconds.

The low and high cognitive workload blocks were randomly and evenly assigned to participants. More specifically, 15 participants assigned an odd ID completed the task in Order 1: low cognitive workload block, high cognitive workload block; and another 15 participants assigned an even ID completed the task in Order 2: high cognitive workload block, low cognitive workload block.

### Procedure

All participants entered the experiment room and performed the experiment once at a time.

First, the participants watched an instructional video of the instruments and experimental procedure (https://youtu.be/_d24CRSwhuQ). They were free to ask any questions after viewing the video.

The experiment started with the participant filling in a demographic questionnaire (Appendix A). This questionnaire covered subjects' age, gender, strong hand, experience with smartphones, and current smartphone usage situation. Then, they wore the MUSE 2 headband and adjusted themselves to a comfortable sitting position. After that, the participants completed an eye-tracking calibration with Tobii Pro Nano followed by 10 s with an eye-open relaxed position and another 10 s with an eye-closed comfortable position.

After the preparation step, the participants completed the training session. They can ask any questions about the N-back task during or after the training section. The training sessions were excluded from data analysis.

Then the participants completed the experiment session of the N-back task at the experiment station, wearing the MUSE 2 headband. They first completed 20 trails of 1-back/2-back stimuli, followed by 20 trails of 2-back/1-back stimuli, with intervals of approximately 1–2 s in between each 1-back/2back stimuli (the time between a response and the display of the next stimuli) and a rest period of 5 s in between 1-back and 2-back blocks. The participants were instructed to respond to tasks as accurately and rapidly as possible. The variation of intervals between each 1-back/2back stimuli caused by the internet loading time varied.

**Figure 4:**
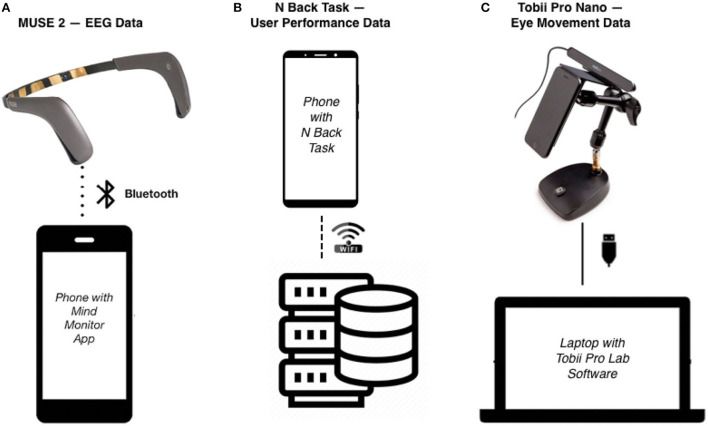
**(A)** The MUSE 2 headband was transmitted over Bluetooth to a phone *via* an application called Mind Monitor for EEG data collection, and the EEG data were uploaded to the research's selected cloud drive after each participant completed the task. **(B)** The N back task was recorded on a website designed by researchers, and the user performance data were collected via the server. **(C)** The Tobii Pro Nano eye tracker was connected to a laptop with Tobii Pro Lab software installed, and the eye movement data were collected by the Tobii Pro Lab software and stored in the hard drive of the laptop.

The MUSE 2 headband collected raw EEG data of TP9, AF7, AF8, and TP10 through an application called Mind Monitor (iOS Version 2.2.0) (Figure 4A). And the Tobii Pro Nano recorded multiple types of eye movement data: pupil dilation, saccade length, saccade velocity, fixation duration, and fixation number (Figure 4B). The self-developed website for the N-back task collected the reaction time and accuracy rate during the experiment (Figure 4C) (N-back task website: http://n-back.artkey.xin/). It is designed for an experiment on a smartphone, and it works best on a smartphone.

Besides the procedure described above, the procedure described in Appendix B when conducting the experiments as a precaution against COVID-19 was followed as well.

### Measures

Data collected from the experiment allow us to examine and compare the following measures:

1. Event-Related Synchronization percentage (ERS) of Theta, Event-Related Desynchronization percentage (ERD) of Alpha, and Event-Related Desynchronization percentage (ERD) (Equation 1).
2. Multiple eye movements: pupil dilation, saccade duration, saccade number in second, fixation duration, and fixation number in second.
3. User performance data: reaction time (RT) and accuracy rate (AR). Reaction time (RT) is the period between the onset of a letter and the response made by a participant. The accuracy rate (AR) is the ratio of the number of correct inputs and the total number of inputs.

## Results, analysis, and discussion

The experiment has investigated the feasibility of using data acquired wirelessly from an EEG headband (MUSE 2) and an eye-tracking device (Tobii Pro Nano) to assess cognitive workload in a well-controlled N-back task in a smartphone setting.

### Demographic data

A total of 30 high school students from Qinghe High School in Huaian, Jiangsu, China completed the experiment, including the demographic questionnaire (Appendix A). Eleven of the 30 participants were female and 19 were male, and the female and male ratio is 11/19. Their average age was 16.34 years old (SD = 0.61). All participants were from the first year in high school. The right hand was the dominant in 29 participants, and the other participant was ambidextrous. The answers to Questions 4–8 of the demographic questionnaire are presented in Table 1. To summarize the data in Table 1, all participants were frequent and proficient smartphone users.

**Table 1:** Participants' answers for Questions 4–8 in the demographic questionnaire.

**Question**	**Answer (*n* = 30)**
Q4: Smartphone: You are able to operate smartphones proficiently.	Strongly agree – 20 Agree – 10 Neither agree nor disagree – 0 Disagree – 0 Strongly disagree – 0
Q5: Smartphone: Which operating system do you use more frequently and proficiently?	iOS – 3 Android – 22 Both – 5 Other – 0
Q6: Smartphone: How many years have you owned/used a smartphone?	< 1 year – 5 1–2 years – 15 3–5 years – 7 5–10 years – 1 >10 years – 2
Q7: Smartphone: How many hours a day on average do you use your smartphone when the school is in session?	0.5–1 h – 28 1–2 h – 2 3–5 h – 0 6–8 h – 0 >8 h – 0
Q8: Smartphone: How many hours a day on average do you use your smartphone when the school is on break?	0.5–1 h – 5 1–2 h – 10 3–5 h – 13 6–8 h – 2 >8 h – 0

### User performance data

Only 29 participants' user performance data were processed, analyzed, and discussed here. Participant ID 126's experimental data were not recorded due to Internet connection issues.

We analyzed user performance data (reaction time and accuracy rate) in order to confirm that participants perceived the various N-Back conditions as different. User performance data processing, user performance data results, and user performance data analysis are included in this section.

#### User performance data processing

The N-Back website recorded participants' ID, N-back order, current letter, participants' choices, accuracy (yes/no/null), reaction time, start timestamp in Unix time, and end timestamp in Unix time. Unix time is a system for describing a point in time and it is the number of seconds that have elapsed since the Unix epoch, excluding leap seconds (Ritchie and Thompson, 1978).

I conducted a Shapiro–Wilk test for the reaction time (RT) of all participants for 1 back and 2 back tests to check its normality. The result is significant (p < 0.001), which indicates that the data are not normally distributed. Therefore, I conducted a Mann–Whitney–Wilcoxon Test for the reaction time (RT) between 1 and 2 back.

#### User performance data results

The descriptive statistics and the Mann–Whitney–Wilcoxon Test results for the reaction time (RT) and accuracy rate (AR) between conditions are presented in Table 2.

**Table 2:** The descriptive statistics and Mann–Whitney–Wilcoxon Test results for reaction time (RT) (unit: second) and accuracy rate (AR) between 1 and 2 back.

**Measure**	**N back**	**N**	**Mean**	**SD**	**Median**	W*	***p*-value**
Reaction time	1	580	1.11	0.35	1.06	91,036	< 2.2e-16***
	2	580	1.58	0.65	1.43		
Reaction time with odd IDs	1	300	1.15	0.32	1.13	25,475	< 2.2e-16***
	2	300	1.65	0.67	1.53		
Reaction time with even IDs	1	280	1.06	0.38	0.98	19,302	< 2.2e-16***
	2	280	1.51	0.62	1.33		
Accuracy rate	1	29	0.94	0.14	1	702	6.547e-06***
	2	29	0.85	0.11	0.9		

* W-Value is the sum of the ranks of the first sample.

*** *P* = 0.001.

#### User performance data analysis

##### Reaction time

The Mann–Whitney U test was conducted to examine whether the reaction time (RT) had statistically significant differences between 1 and 2 back for all participants, for participants with odd IDs, and for participants with even IDs. The *p*-values (< 0.001) indicate the answer is yes, as expected (Table 2).

##### Accuracy rate

The Mann–Whitney–Wilcoxon test was conducted to examine whether the accuracy rate (AR) had significant differences between 1 back and 2 back for the 29 participants. The p-value (<0.001) indicates the answer is yes, as expected. The median of 1 back accuracy rate is 1, which is higher than the median of 2 back accuracy rate (0.9) (Table 2).

#### User performance data discussion

As expected, the reaction time (RT)increased and accuracy rate (AR) decreased with the leveled-up differential of N-Back tasks, and 1 and 2 back tasks did create low and high cognitive workload conditions for the experiment's participants.

### EEG data

#### EEG data processing and analysis

Only 29 participants' EEG data were processed, analyzed, and discussed here. Participant ID 126's EEG was not recorded due to Internet connection issues.

During the experiment, participants wore a MUSE 2 headband connected to the Mind Monitor. The Mind Monitor collected their EEG data. According to the Technical Manual from the Mind Monitor website, bandpass filtering was carried out on the raw data with power noise at 50 Hz or 60 Hz. Then, a fast Fourier transform (FFT) calculation (Heckbert, 1995) was applied to the raw data to get Theta, Alpha, and Beta.

The recorded EEG signals were processed using Excel and R to get two baselines:

- baseline_near: Based on the timestamps recorded by the N-back task website, we sectioned the intervals starting from−200 ms to the onset of each letter as the baseline interval for each letter (Xiang et al., 2021).
- baseline_away: According to the timestamps recorded by the N-back task website, we segmented the first 3,000 ms of the 10 s relaxing eyes open relaxing as a baseline.

Computed ERD of Alpha {TP9, AF7, AF8, TP10}, ERD of Beta{TP9, AF7, AF8, TP10}, and ERS of Theta {TP9, AF7, AF8, TP10} with baseline near for each letter interval; and ERD of Alpha AF7, Alpha AF8, Beta AF8, and Beta TP9 with baseline away for each letter interval. The interval starts from the onset of each letter to the time point that a choice is being made by participants, which is definitely ≤ 3 s.

A non-parametric test, the Mann–Whitney–Wilcoxon test, was selected for non-normal data. The Mann–Whitney–Wilcoxon test was conducted for 12 measures (Alpha {TP9, AF7, AF8, TP10}, ERD of Beta{TP9, AF7, AF8, TP10}, and ERS of Theta {TP9, AF7, AF8, TP10} with baseline_near between 1 back and 2 back; and ERD of Alpha AF7, Alpha AF8, Beta AF8, and Beta TP9 with baseline away 1 back and 2 back. See Appendix C for the details.

#### EEG data results

The average workload is the average value of instantaneous loads within a task duration (Xie and Salvendy, 2000). In this study, the average cognitive workload is represented by the average ERD of Alpha, Beta, and ERS of Theta for TP9, TP10, AF7, and AF8 of the intervals of each letter with baseline_near.

To examine whether there is a difference between 1 and 2 back for averages of ERD of Alpha, Beta, and ERS of Theta for TP9, TP10, AF7, and AF8 with baseline near, we conducted Mann–Whitney–Wilcoxon tests between 1 and 2 back of all participants. The results are included in Table 3. Due to computational problems, the numbers of subjects (N) vary between the analyses.

**Table 3:** The descriptive statistics and Mann–Whitney–Wilcoxon test results for averages of ERD of Alpha, Beta, and ERS of Theta for TP9, TP10, AF7, and AF8 with baseline near between 1 and 2 back.

**Measure**	**N Back**	**N**	**Mean**	**SD**	**Median**	W*	***p*-value**
Theta_ERS_TP9_near	1	415	0.5103788	2.3437866	0.1606055	81,290	0.05664
	2	424	0.3624826	0.8789823	0.1961127		
Theta_ERS_AF7_near	1	442	1.479168	7.528482	0.3261652	102,550	0.1951
	2	488	40.222121	858.424241	0.3993164		
Theta_ERS_AF8_near	1	463	256.550957	4473.14064	0.4962916	107,560	0.7199
	2	471	1.656752	6.897568	0.4979768		
Theta_ERS_TP10_near	1	402	0.1968689	0.2983741	0.1055942	76,520	0.1935
	2	402	0.4827313	2.7181894	0.1440152		
Alpha_ERD_TP9_near	1	407	−0.2429342	0.6022346	−0.0708803	94,305	0.07823
	2	433	−1.0891115	16.1080866	−0.0994483		
**Alpha_ERD_AF7_near**	1	417	−0.4870307	1.31401	−0.1177238	89,413	0.03469**
	2	395	−4.1949784	63.64311	−0.1726443		
Alpha_ERD_AF8_near	1	422	−1.305807	7.191686	−0.1905496	91,227	0.9368
	2	431	−7.442734	122.014022	−0.2087486		
Alpha_ERD_TP10_near	1	382	−0.3358805	1.1701908	−0.0741198	75,913	0.6915
	2	391	−0.2689888	0.5359719	−0.0839877		
**Beta_ERD_TP9_near**	1	424	−0.0948762	0.1298309	−0.053213	94,695	0.04122**
	2	413	−0.1438368	0.3410383	−0.0645524		
Beta_ERD_AF7_near	1	398	−0.4218005	1.783285	−0.0801937	86,126	0.8545
	2	436	−0.6272942	3.536376	−0.0829866		
Beta_ERD_AF8_near	1	431	−0.1898465	0.3919714	−0.0811931	99,618	0.1104
	2	435	−0.2896695	0.7094699	−0.1054581		
Beta_ERD_TP10_near	1	259	−0.1434616	0.2283444	−0.0740742	36,067	0.2439
	2	263	−0.1571087	0.1960775	−0.0797461		

* W-Value is the sum of the ranks of the first sample. The bold values are *p* values smaller than 0.01, so they are statistically significantly.

** *P* = 0.01.

It is not feasible to have a baseline for each stimulus in scenarios of users experiencing an application on smartphones. Hence, to explore the feasibility of adopting a single baseline away with stimulus, we conducted Mann–Whitney–Wilcoxon tests for averages of ERD of Alpha AF7 and Beta TP9 with baseline away between 1 and 2 back for all participants, participants with odd IDs, and participants with even IDs, respectively. We find no significant results (Table 4).

**Table 4:** The descriptive statistics and Mann–Whitney–Wilcoxon test results for averages of ERD of Alpha_AF7 and Beta_TP9 with baseline away between 1 back and 2 back.

**ID**	**Measure**	**N Back**	**N**	**Mean**	**SD**	**Median**	W*	***p*-value**
ALL	Alpha_ERD_AF7_away	1	325	−0.707954	0.6306785	−0.548365	56,233	0.2634
		2	364	−0.707059	0.7387755	−0.4852188		
	Beta_ERD_TP9_away	1	179	−0.119261	0.1193088	−0.0843989	20,950	0.1648
		2	205	−0.154169	0.1637146	−0.1034079		

* W-Value is the sum of the ranks of the first sample.

#### Analysis of EEG results

Different cognitive workloads evoked associated human brain oscillatory responses (Krause et al., 2000; Pesonen et al., 2007) that made it possible to measure the corresponding cognitive workload levels.

The results in Table 3 show that only Alpha ERD AF7 and Beta ERD TP9 among the 12 measures (TP9, AF7, AF8, TP10 ^*^ Alpha, Beta, and Theta) are sensitive to the different workloads of between 1 and 2 back conditions for all participants (p < 0.05).

**Figure 5:**
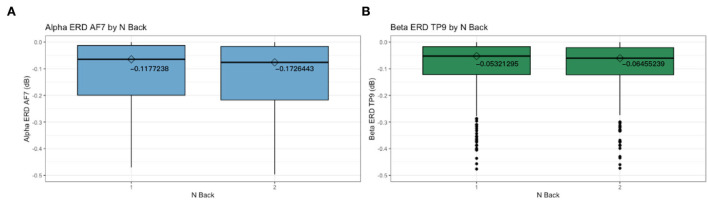
Box plots with medians between 1 back and 2 back for Alpha ERD AF7 **(A)** and Beta ERD TP9 **(B)**.

The magnitudes of Alpha ERD AF7 and Beta ERD TP9 are significantly greater for the 2 back than for the 1 back (Figure 5), indicating that Alpha and Beta increase as tasks demand more cognitive workload. This is in line with previous studies that found with inclining task demands, Alpha and Beta desynchronize (increase) (Klimesch, 1999; Stipacek et al., 2003; Klimesch et al., 2005; Neubauer et al., 2006; Antonenko et al., 2010a; Antonenko and Niederhauser, 2010b; Xiang et al., 2021).

The insensitivity of the six measures (AF8 and TP10 ^*^ Alpha, Beta, and Theta) can be explained by the functions of the cerebral cortex.

The TP10 electrode is positioned behind the right ear, which is the right temporal lobe, and the AF8 electrode is on the right forehead, which is the right frontal lobe (Figure 1). The TP9 electrode is positioned behind the left ear, which is the left temporal lobe, and the AF7 electrode is on the left forehead, which is the left frontal lobe (Figure 1).

The left temporal lobe is associated with understanding language, learning, memorizing, forming speech, and remembering verbal information (Guy-Evans, 2021b). We used English letters as a stimulus in the N-back task and participants' primary language is Chinese, thus it makes sense that Beta ERD TP9 are found to have significant differences between 1 and 2 back.

The AF7 electrode is on the left side of the frontal lobe. The frontal lobe is located behind the forehead, at the front of the brain. Each lobe controls the operations on opposite sides of the body: the left hemisphere controls the right side of the body and vice versa (Guy-Evans, 2021a). It is believed that the left frontal lobe works predominantly with language, logical thinking, and analytical reasoning. The right frontal lobe, on the other hand, is mostly associated with non-verbal abilities, creativity, imagination, musical, and art skills (Guy-Evans, 2021a).

The dominant hands of 29 participants were the right hand, and the remaining one was both hands. We observed all participants only used their right hands to make the choices. This explains that the Alpha ERD AF7 (left forehead) but not AF8 (right forehead) was found to have significant differences between 1 and 2 back. However, the other four measures of TP9 and AF7 shall be sensitive to the difference in cognitive workload, as previous studies prove.

#### Discussion on EEG data

In summary, MUSE 2 outputs good signals, but these signals may not be readily useful in the studies on the usability of smartphone applications for an entire and consecutive user experience as a result of the difficulty in selecting a sensible baseline due to two rationales.

The first rationale behind the difficulty in the selection of a sensible baseline lies in the fact that only Alpha ERD AF7 and Beta ERD TP9 show sensitivity to the difference in cognitive workload between 1 and 2 back. It is not consistent with that given in previous studies (Klimesch, 1999; Stipacek et al., 2003; Klimesch et al., 2005; Neubauer et al., 2006; Antonenko and Niederhauser, 2010b; Xiang et al., 2021).

Second, according to Pfurtscheller and Lopes da Silva (1999), ERD/ERS is required to have a baseline captured some seconds right before the events. Yet, it is not feasible to have a baseline for each stimulus in the scenarios of users experiencing any applications on smartphones. We had carried out an exploration of adopting a single baseline away with stimulus, but unfortunately it did not show any statistically significant differences for averages of ERD of Alpha AF7 and Beta TP9 between 1 and 2 back.

All in one sentence, although MUSE 2 is of consumer grade, comfortable to wear, and wireless connected, it is a reliable device for researchers to capure stable EEG data for measuring cognitive workload. It does show some promise for detecting cognitive workload elicited by isolated/independent elements in user interface (UI) design, and selective signals may be combined with eye-tracking data to detect UI issues that invoke user errors.

### Eye movement data

#### Eye movement data processing and analysis

During the experiment, participants completed N-back tasks on a smartphone attached to the mobile testing accessory with the Tobii Pro Nano mounted on the top (Figure 2). Eye movement data were collected by the Tobii Pro Nano *via* the Tobii Pro Lab software (version 1.162.32461).

Similar to the EEG data, the recorded eye movement data were processed using Excel and R through several steps to get averaged pupil dilation left, averaged pupil dilation right, averaged fixation duration, averaged fixation number in second, averaged saccade duration, averaged saccade number in second, maximums of pupil dilation left, maximums of pupil dilation right, maximums of fixation duration, and maximums of saccade duration.

A non-parametric test was selected for non-normal data. See Appendix C for data processing and analysis detailed steps.

#### Eye movement data results

The Tobii Pro Nano was extremely sensitive to angle changes between participant's eyes and the device. Based on the experience we had gathered from pilots, we had a higher chair for the participants to improve the capture rate and adjusted the Tobii Pro Nano angle according to each participant. Despite the higher chair we had employed and the active adjustments made to the mobile testing accessory, the capture rates varied across participants. Eighteen participants' data with relatively higher capture rates were processed and analyzed here.

Averaged cognitive workload is quantified by the averaged pupil dilation left and the averaged pupil dilation right, the averaged fixation duration and the averaged fixation number, and the averaged saccade duration and the averaged saccade number.

To examine whether there were statistically significant differences for averaged pupil dilation changes between low and high cognitive workload conditions, we conducted a Mann–Whitney–Wilcoxon test for averaged pupil dilation left, averaged pupil dilation right, between 1 and 2 back. The results are presented in Table 5.

**Table 5:** The descriptive statistics and Mann–Whitney–Wilcoxon test results for the averaged pupil dilation left, the averaged pupil dilation right, the averaged fixation duration, the averaged fixation number, the averaged saccade duration, and the averaged saccade number in second between 1 and 2 back.

**Measure [mm]**	**N back**	**N**	**Mean**	**SD**	**Median**	W*	***p*-value**
Averaged pupil dilation left	1	245	0.0776042	0.3685327	0.1129762	24,874	0.0006402***
	2	247	0.2135481	0.3894946	0.1967991		
Averaged pupil dilation right	1	255	0.1306679	0.4081041	0.1599511	29,682	0.005187**
	2	271	0.2631821	0.4403823	0.2407292		
Averaged fixation duration [ms]	1	247	224.4167	186.5857	166.7222	30,689	0.6749
	2	254	217.8477	167.6373	166.4935		
**Fixation number in second**	1	247	25.89081	15.4502	22.97702	33,166	0.02676**
	2	254	24.25003	14.94318	22.54331		
Averaged saccade duration[ms]	1	267	27.14384	10.29906	25	37,158	0.9221
	2	277	27.43823	13.47225	25		
**Saccade number in second**	1	267	11.416636	6.600944	10.673235	41,258	0.0196**
	2	277	9.862231	5.004347	9.687836		

* W-Value is the sum of the ranks of the first sample. The bold values are *p* values smaller than 0.01, so they are statistically significantly.

** *P* = 0.01;

*** *P* = 0.001.

In this research, the averaged fixation duration and the fixation number in second were adopted as the representative of the averaged cognitive workload during the intervals, starting from the appearance of each letter to the time point that choices were made.

To examine whether there were statistically significant differences for averaged fixation duration and fixation number in second between low and high cognitive workload conditions were observed, we conducted Mann–Whitney–Wilcoxon tests between 1 and 2 back. The results are presented in Table 5.

In this research, the averaged saccade duration and the averaged saccade number in second were adopted as the representative of the averaged cognitive workload during the intervals, starting from the appearance of each letter to the time point that choices were made.

To examine whether there were statistically significant differences for the averaged saccade duration and the averaged saccade number in second between low and high cognitive workload conditions, we conducted Mann–Whitney–Wilcoxon tests between 1 and 2 back. The results are presented in Table 5.

The maximum pupil dilation left and the maximum pupil dilation right were adopted as the representative of the peak cognitive workload during the intervals, starting from the appearance of each letter to the time point that choices were made.

To examine whether statistically significant differences for maximums of pupil dilation changes between low and high cognitive workload condition, we conducted Mann–Whitney–Wilcoxon tests for maximums of pupil dilation left and of pupil dilation right between 1 and 2 back. The results are presented in Table 6.

**Table 6:** The descriptive statistics and Mann–Whitney–Wilcoxon test results for the maximum pupil dilation left and the maximum pupil dilation right between 1 and 2 back.

**Measure [mm]**	**N back**	**N**	**Mean**	**SD**	**Median**	W*	***p*-value**
**Maximums of pupil dilation left**	1	299	−0.5025064	1.676639	0.1764088	26,852	0.03083**
	2	300	−0.3933013	1.735202	0.2990345		
**Maximums of pupil dilation right**	1	300	−0.30043015	1.54833	0.2411331	37,104	0.0001998***
	2	300	0.04463652	1.391381	0.397199		

* W-Value is the sum of the ranks of the first sample.

** *P* = 0.01;

*** *P* = 0.001.

In this research, the maximums of fixation duration were adopted as the representative of the peak cognitive workload during the intervals, starting from the appearance of each letter to the time point that choices were made.

To examine whether statistically significant differences exist for the maximums of fixation duration between low and high cognitive workload conditions, we conducted Mann–Whitney–Wilcoxon tests between 1 and 2 back. We find no significant results (Table 7).

**Table 7:** The descriptive statistics and Mann–Whitney–Wilcoxon test results for the maximums of fixation duration between 1 and 2 back for all selected participants.

**Measure**	**N Back**	**N**	**Mean**	**SD**	**Median**	W*	***p*-value**
Maximum of fixation duration [ms]	1	247	290.8664	226.5789	217	30234	0.3964
	2	256	309.6367	247.3612	217		
Maximum of saccade duration[ms]	1	267	42.42697	23.30892	33	36237	0.5242
	2	280	43.71071	24.47466	33		

* W-Value is the sum of the ranks of the first sample.

In this research, the maximum saccade duration was adopted as the representative of the peak cognitive workload during the intervals, starting from the appearance of each letter to the time point that choices were made.

To examine whether statistically significant differences exist for the maximums of saccade duration between low and high cognitive workload conditions, we conducted Mann–Whitney–Wilcoxon tests for it between 1 and 2 back. We find no significant results (Table 7).

#### Eye movement results' analysis

Overall, the eye movement data collected by the Tobii Pro Nano are valid and reliable. Some measures (pupil dilation, saccade number in second, fixation number in second) are sensitive to the difference of average cognitive workload and peak cognitive workload introduced by the 1 or 2 back tasks.

The averages of pupil dilations of both eyes have been proven to be reactive to the differences of average cognitive workload between 1 and 2 back tasks consistently. As Table 5 reveals, there are statistically significant differences between 1 and 2 back for the averages of pupil dilation of both eyes (*p* < 0.05). The medians of the averages of pupil dilations of both eyes are larger in 2 back than in 1 back. The medians of the averages of pupil dilations of both eyes remain greater in the 2 back and in the 1 back, so that the bigger average of pupil dilations means a higher averaged cognitive workload. This finding is in line with earlier studies (Granholm et al., 1996; Pomplun and Sunkara, 2003; Klingner et al., 2008; Chen et al., 2011; Porta et al., 2012; Rafiqi et al., 2015).

The same pattern is discovered in the maximums of pupil dilation. There are statistically significant differences between 1 back and 2 back for maximums of pupil dilation of both eyes (*p* < 0.05 or < 0.001) (Table 6). The medians of the maximum pupil dilation of both eyes are larger in 2 back than in 1 back for both eyes, which indicates the larger maximum of pupil dilations means a higher peak cognitive workload.

As for fixation and saccade, statistically significant differences were observed between 1 and 2 back in the fixation number in second and saccade number in second (Table 6). Irreconcilable with previous findings is that the correlation between the number of fixation and cognitive workload is negative. Previous studies have concluded that an upswing number of fixations correlate with an increased cognitive load level (Goldberg and Helfman, 2010; Chen et al., 2011; Wang et al., 2014; Zagermann et al., 2018). And the higher number of saccades in second (saccade velocity) is also related to lower cognitive workload, opposing the previous research (Barrios et al., 2004; Chen et al., 2011; Lallé et al., 2016; Zagermann et al., 2018).

#### Discussion on eye movement data

In this study, we found that eye-tracking device, Tobii Pro Nano with mobile testing accessory, appears to be a valid instrument for monitoring the cognitive workload difference in a smartphone setting. This finding along with previous studies (Sugaya, 2019; Ehlers, 2020; Lee and Chenkin, 2020) can provide an initial empirical evidence on the reliability of Tobii Pro Nano with mobile testing accessory. Moreover, the average pupil dilation and the maximum pupil dilation have been ratified as the effective measures of cognitive workload difference in a smartphone setting, and they enlarge along with the difficulty levels of N-back task rising.

One incongruent finding is that the fixation velocity and saccade velocity decline with the increment of cognitive workload, while the previous studies found an upswing number of fixations correlate with an increased cognitive load level (Barrios et al., 2004; Goldberg and Helfman, 2010; Chen et al., 2011; Wang et al., 2014; Lallé et al., 2016; Alonso Dos Santos and Calabuig Moreno, 2018; Zagermann et al., 2018).

One possible justification for this reverse is the different task design. The N-back task only required participants to look at one spot on the screen, while the previous studies required participants to observe, scan, and search during tasks and the gazes were not fixed in one spot (Table 8).

**Table 8:** The tasks in the papers.

**Paper**	**Task**
Chen et al. (2011)	observing team player positions in basketball game videos
Goldberg and Helfman (2010)	scanning within and between bar, line, and spider graphs
Barrios et al. (2004)	browsing content to learn
Lallé et al. (2016)	retrieve, find, sort, and compute in charts
Wang et al. (2014)	online shopping tasks on a shopping website
Zagermann et al. (2018)	three visual search tasks that represent different levels of difficulty

Another obvious concern about the Tobii Pro Nano is the unstable capture rate. Only nearly half of the participants' data was captured enough to be adopted.

One pilot participant's capture rate was 0% and he mentioned that he had a high degree of astigmatism, around 500–600 in both eyes. Astigmatism is an imperfection in the curvature of your eye's cornea or lens (Boyd, 2021). It may be helpful to think of the normal eye as being shaped like a basketball. With astigmatism, it is shaped more like an American football. The Tobii Pro Nano may not effectively recognize the eyes of people with astigmatism. This suggests that the low capture rate for some participants may be caused by astigmatism. Therefore, information about astigmatism was obtained from the participants.

**Figure 6:**
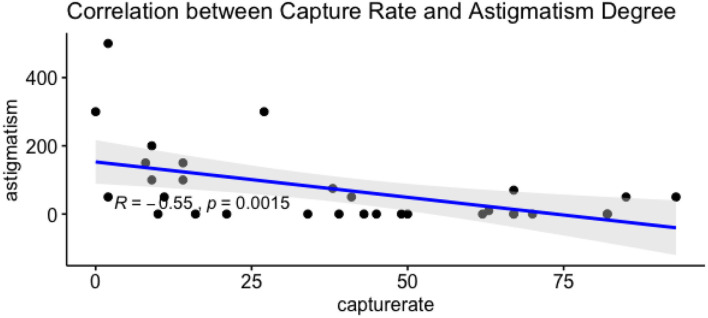
The correlation between capture rate and astigmatism degree.

For the astigmatism degree, we averaged two eye degrees. The capture rate was recorded in the Tobii Pro Nano. We adopted Spearman's rho statistic to assess the correlation between capture rate and astigmatism degree, and the correlation coefficients and p values are given in Figure 6. The result shows that there is a statistically significant negative correlation between capture rate and astigmatism degree (*p* < 0.05 or < 0.001). The correlation coefficient is −0.55. The negative correlation between astigmatism and capture rate may have resulted from the changes in the shape of eyeballs.

Overall, the objective of Experiment 1 was to assess the feasibility of using wirelessly acquired EEG (MUSE 2) and eye-tracking device (Tobii Pro Nano) to assess cognitive workload in a well-controlled N-back task in a smartphone setting. And the eye-tracking device, Tobii Pro Nano, can be adopted as a device to collect eye movement data to monitor cognitive workload fluctuations in a smartphone setting with a screen for high astigmatism, and pupil dilation can be measured for cognitive workload differences.

## Conclusion and future directions

This study aimed to verify the feasibility of using eye-tracking (i.e., Tobii Pro Nano) and low-cost electroencephalogram (EEG, i.e., MUSE 2) devices to measure real-time cognitive workload changes during mobile application use, and which measures are sensitive to cognitive workload differences. Results from the experiment manifest that the eye-tracking device (Tobii Pro Nano) can be adopted as a device to collect eye movement data to monitor cognitive workload fluctuations in a smartphone setting, and pupil dilations can be used to measure the cognitive workload differences, with a screening test to filter out people with a high astigmatism degree.

There are three main directions for future research. The first one is to adopt pupil dilations as an effective measure to assess users' cognitive workloads while experiencing a smartphone application and to improve the UI design of the application based on the assessment.

The second one is to expand the age range to cover middle-aged and older adults. Only younger users who were born with smartphones were included in the study and we found that they have a high level of endurance for design issues and proficient capability to resolve issues by themselves. While it has been found that cognitive performance declines with age (Deary et al., 2009), it is reasonable to expand research to include middle-aged and older adults to verify the findings in the different age groups to investigate whether experience with smartphones overcomes cognitive ability's recession.

The third one is to test findings in other settings, e.g., virtual reality (VR), wearable devices, etc. Some ubiquitous screens (e.g., smart watches, etc.) and certain brand immersive experience technologies (e.g., VR glasses, etc.) have been winning consumers' heart with acceptable prices and great user experiences. It is necessary to test our findings in these settings as well.

In the far future, one major direction we desire to explore is to establish a multidimensional assessment tool for product usability, including subjective ratings, psychophysiological measures, and performance measures. We understand this objective is extensive and requires considerable time and human resources to complete.

Another direction is to expand from application-focused studies to include cognitive-focused studies. Instead of studying how to improve the usability of specific kinds of software applications, we aim to study cognitive processes, such as how to help people focus or refocus in different settings.

## Data availability statement

The raw data supporting the conclusions of this article will be made available by the authors upon request.

## Ethics statement

The studies involving human participants were reviewed and approved by University of Arizona IRB Office (Protocol Number: 2101428836). Written informed consent to participate in this study was provided by the participants' legal guardian/next of kin.

## Author contributions

Study conception and design, data collection, and draft manuscript preparation: LZ. Analysis and interpretation of results: LZ and HC. All authors reviewed the results and approved the final version of the manuscript.

## Funding

NSF award # 1661485 and University of Arizona SBS summer dissertation fellowship.

## Conflict of interest

The authors declare that the research was conducted in the absence of any commercial or financial relationships that could be construed as a potential conflict of interest.

## Publisher's note

All claims expressed in this article are solely those of the authors and do not necessarily represent those of their affiliated organizations, or those of the publisher, the editors and the reviewers. Any product that may be evaluated in this article, or claim that may be made by its manufacturer, is not guaranteed or endorsed by the publisher.
