# Supplementary material for: Reliability of MUSE 2 and Tobii Pro Nano at capturing mobile application users' real-time cognitive workload changes
Source: Front Neurosci. 2022 Nov 28;16:1011475. doi: 10.3389/fnins.2022.1011475 (PMC9743809; doi:10.3389/fnins.2022.1011475)
Supplement: Supplementary file 1 [file Data_Sheet_1.docx]

Appendix A : Demographic questionnaire

1. Your ID: ___ (Each subject will be given an 3-digit ID.)
2. Age: What is your age? ____
3. Gender: What is your gender?

Male

Female

Transgender

Other

Prefer not to respond

1. Which hand is your strong hand?

Left

Right

Both

1. You are able to operate smart phones proficiently.

Strongly agree, Agree, Neither agree nor disagree, Disagree, Strongly disagre

1. Which operating system are you more familiar with?

iOS system

Android system

Both

Other:______

1. How many year since you own your first smartphone?

Less than one year

1-2 years

3-5 years

5-10 years

More than 10 years

1. How many hours a day on average do you use your smartphone during school session?

0.5-1 hour

1-2 hours

3-5 hours

6-8 hours

More than 8 hours

1. How many hours a day on average do you use your smartphone during school break?

0.5-1 hour

1-2 hours

3-5 hours

6-8 hours

More than 8 hours

# Appendix B : Guidelines for COVID-19

To minimize the risks posed by COVID-19, We will follow the guidelines provided by the IRB office on mitigating risk. Specifically we will follow the procedure described below when conducting the experiments (updated from the RII checklist):

1. The room where the experiment will be conducted contains one desk, one chair, one computer mounted with an eye tracker, two EEG devices, one basket holding sanitation materials and clean face masks, and one wastebasket with cover.
2. Schedule subjects so that there is 15 mins of cleaning up time in between any two subjects.
3. Subjects are screened by phone using<https://arizona.app.box.com/s/a5l3s9q9t12bogiswbnhxn2rrgcyg9uq> before coming to the study. Subjects are informed that there is no waiting area so they should come at their scheduled time.
4. Researchers disinfect all surfaces before a subject comes into the room.
5. Subject comes in.
6. Subject uses hand sanitizer, and puts on a face mask provided.
7. Researcher uses hand sanitizer, and puts on a face mask. Researchers conduct the same wellness screening again with the subject. If not passed, the subject is sent home. Otherwise, continue:
8. Play recorded script: https://arizona.app.box.com/s/u9p45rattnquqnce06ppzkf1on49ashl
9. Researcher sets up the experiment with the subjects via verbal instructions from 6 feet away.
10. Researcher uses hand sanitizer and leaves the subject and moves more than six feet away or into another room.
11. Subject completes the task on the computer/smartphone. Subject has access to hand sanitizer at all times.
12. Still being six feet away or in another room, researchers conduct the 10 min interview on Zoom with the subject (to reduce contact).
13. Subjects use hand sanitizer, leave the experiment room, and drop out the masks in a bin right outside the experiment room.
14. Researcher changes a new mask, drops the face mask in the waste bin, spray 15% bleach in the waste bin, then sanitizes all the surfaces again.
15. Let the next subject in.

In addition, high risk population will not be recruited for the research: all persons age 65 or older, or persons of any age with a medical condition including lung disease or moderate-severe asthma, serious heart condition, immune system compromise, obesity (BMI >39), diabetes, or chronic kidney or liver disease.

No more than one research participant to be present in the study area at a given time.

All research staff should self-screen daily using the University of Arizona COVID-19 screening https://arizona.app.box.com/s/a5l3s9q9t12bogiswbnhxn2rrgcyg9uq.  Researchers who are experiencingCOVID-19 symptoms or have been exposed to individuals with COVID-19 should refrain from engaging in face-to-face human research activities for 14 days.

# Appendix C: Processed steps

The recorded EEG signals were processed using Excel and R through the steps below:

1. Set the “TimeStamp” cell format to yy/m/d hh:mm:ss.000, and one column ID (the three-digits participants were assigned), was added to the CSV file,
2. Dropped all data points with bad connections (4 for HIS)；
3. Dropped all data points that were classified as elements,  such as blink, jaw_clench, etc.
4. Based on the timestamps recorded by the N-back task website, we sectioned the intervals starting from -200ms (approximately*) to the onset of each letter as the baseline interval for each letter (Xiang et al., 2021). Averaged these intervals to get the baseline for each letter. This baseline is called baseline_near, and refers to it being near the stimulus . The reason that the time windows are not exactly 200ms is that the EEG data is recorded at 256 Hz (256 times per second) so that it is not eligible to have a full 200ms time window. Therefore, we selected the time points that are most closely aligned.
5. Based on the timestamps recorded by the N-back task website, we sectioned the intervals starting from the appearance of each letter to the time point that choices were made, and filtered out the duration of blanks . Columns  N-back, Letter Order) were added. N-back contained 1 or 2 back.  Letter order was the orders of letters in 1 or 2 back, it ranged from 1 to 20.

1. Calculated ERD/ERS with baseline_near , then these columns (Theta_E_{TP9, AF7, AF8, TP10}, Alpha_E_{TP9, AF7, AF8, TP10}, Beta_E_{TP9, AF7, AF8, TP10}, ) are added.
2. Separate ERD and ERS based on their signs (+ or -).
3. Averaged each letter interval for ERD of Alpha_E_{TP9, AF7, AF8, TP10}, Beta_E_{TP9, AF7, AF8, TP10}, and ERS of Theta_E_{TP9, AF7, AF8, TP10}.
4. Conducted Mann-Whitney-Wilcoxon tests for averages of ERD of Alpha_{TP9, AF7, AF8, TP10}, Beta_{TP9, AF7, AF8, TP10}, and ERS of Theta_{TP9, AF7, AF8, TP10}between low cognitive workload (1 back) and high cognitive workload (2 back) for all participants.
5. Conducted Mann-Whitney-Wilcoxon tests for averages of ERD of Alpha_{TP9, AF7, AF8, TP10}, Beta_{TP9, AF7, AF8, TP10}, and ERS of Theta_{TP9, AF7, AF8, TP10} between low cognitive workload (1 back) and high cognitive workload (2 back) for participants with odd IDs, and participants with even IDs, respectively.
6. Then according to the timestamps recorded by the N-back task website, we segmented the first 3000ms of the 10s relaxing eyes open relaxing as baseline. This baseline is called baseline_away, and refers to it being far away from the stimulus.
7. Calculated ERD/ERS of Alpha AF7, Alpha AF8,  Beta AF8  and Beta TP9 with baseline_away, then four columns (Alpha_AF7_E_2, Alpha_AF8_E_2 , Beta_AF7_E_2, Beta_TP9_E_2) were added.
8. Separate ERD and ERS based on their signs (+ or -)
9. Averaged each letter interval for ERD of Alpha_AF7_E_2, Alpha_AF8 _E_2,   Beta_AF8_E_2 and Beta_TP9_E_2 .
10. Conducted Mann-Whitney-Wilcoxon tests for averages of ERD of Alpha_AF7_E_2 and Beta_TP9_E_2 between low cognitive workload (1 back) and high cognitive workload (2 back) for all participants, participants with odd IDs, and participants with even IDs, respectively.

The collected eye movement data were processed using Excel and R. Similar to EEG data, the recorded eye movement data were processed using Excel and R through these steps:

1. Based on the timestamps recorded by the N-back task website and the recording start time marked by Tobii Pro Lab, we calculated the intervals in milliseconds of the low cognitive workload block and the high cognitive workload block for each participant, and the intervals in milliseconds of each wrong and missing response.
2. Mapped these intervals to the eye movement data according to the column “Recording Timestamp '', and added three new columns: N back (1, 2) ,
3. After the preprocessing of EEG data by 2 steps, we had a data frame with 208261 entries and 39 variables. Check the Eye movement type columns,  and it has four types:

EyesNotFound     Fixation      Saccade    Unclassified

126171                 39367         20916        21807.

Figure 1 demonstrates the eye movement type distribution across participants. As a result of  the high percentages of eyes not captured, the data of participants (ID: 101,103, 106,112,114,115,117,120,121,122,123,124,128,129) were dropped out. A total of 14 participants (ID: 102,104,107, 108,109,110,111,113,116,118,119,125,126,130) were processed and analyzed further.


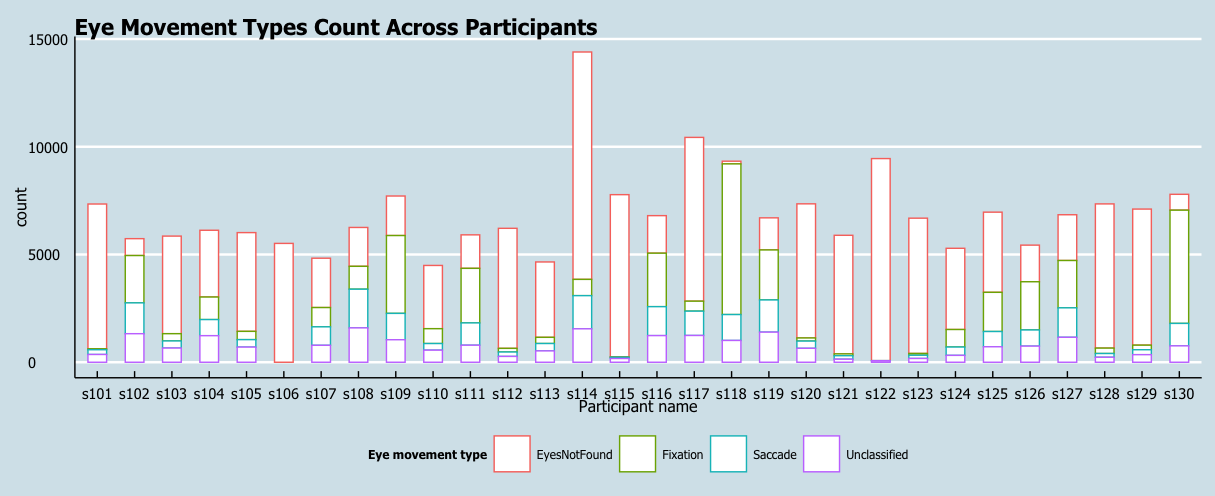


Figure 1: Eye movement type distribution across participants.

1. Based on the timestamps recorded by the N-back task website and the recording start time marked by Tobii Pro Lab, we sectioned the eye-tracking data for the baseline for pupil diameters of the first 3s of the 10s eye open relaxation. Averaged the 3s intervals to get the baseline of pupil diameter.
2. Based on the timestamps recorded by the N-back task website and the recording start time marked by Tobii Pro Lab, we sectioned the eye-tracking data for the intervals starting from the appearance of each letter to the time point that choices were made.
3. Selected these columns: ID, N-back, Pupil diameter left, Pupil diameter right, fixation duration, fixation number, saccade duration, saccade number.

1. Averaged each interval to get averaged Pupil diameter left, averaged Pupil diameter right, averaged fixation duration, averaged fixation number, averaged saccade duration, averaged saccade number.
2. Computed Pupil dilation left and Pupil dilation right by the pupil diameter minus the pupil diameter baseline.
3. Divided the averaged fixation number and the averaged saccade number by the reaction time (unit: second) for each letter of each participant.
4. Averaged Pupil dialtion right between low cognitive workload (1 back) and high cognitive workload (2 back). Then I conducted the Mann-Whitney-Wilcoxon test for averaged pupil dilation left, averaged pupil dilation right between 1 back and 2 back for participants with odd IDs, and for participants with even IDs, respectively.
5. Conducted the Mann-Whitney-Wilcoxon test for averaged fixation duration, averaged fixation number between 1 back and 2 back. Then I conducted the Mann-Whitney-Wilcoxon test for averaged fixation duration, averaged fixation number between 1 back and 2 back for participants with odd IDs, and for participants with even IDs, respectively.
6. Conducted the Mann-Whitney-Wilcoxon test for averaged saccade duration, averaged saccade number between 1 back and 2 back.  Then I conducted the Mann-Whitney-Wilcoxon test for averaged saccade duration, averaged saccade number between 1 back and 2 back for participants with odd IDs, and for participants with even IDs, respectively.
7. Selected the maximums of each interval to get maximums of  pupil dilation left, maximums of pupil dilation right, maximums of fixation duration, and maximums of saccade duration. Then repeat steps 10,11,12 for the maximums.
